# Digital Quantification of Tumor PD-L1 Predicts Outcome of PD-1-Based Immune Checkpoint Therapy in Metastatic Melanoma

**DOI:** 10.3389/fonc.2021.741993

**Published:** 2021-09-21

**Authors:** Jan-Malte Placke, Camille Soun, Jenny Bottek, Rudolf Herbst, Patrick Terheyden, Jochen Utikal, Claudia Pföhler, Jens Ulrich, Alexander Kreuter, Christiane Pfeiffer, Peter Mohr, Ralf Gutzmer, Friedegund Meier, Edgar Dippel, Michael Weichenthal, Lisa Zimmer, Elisabeth Livingstone, Jürgen C. Becker, Georg Lodde, Antje Sucker, Klaus Griewank, Susanne Horn, Eva Hadaschik, Alexander Roesch, Dirk Schadendorf, Daniel Robert Engel, Selma Ugurel

**Affiliations:** ^1^Department of Dermatology, University Hospital Essen, University of Duisburg-Essen, Essen, Germany; ^2^Institute of Experimental Immunology and Imaging, Department of Immunodynamics, University Hospital Essen, Essen, Germany; ^3^Department of Dermatology, Medical Hospital, Erfurt, Germany; ^4^Department of Dermatology, University of Lübeck, Lübeck, Germany; ^5^Department of Dermatology, Venerology, and Allergology, University Medical Center, Ruprecht-Karls University of Heidelberg, Mannheim, Germany; ^6^German Consortium of Translational Cancer Research (DKTK), German Cancer Research Center (DKFZ), Heidelberg, Germany; ^7^Department of Dermatology, Saarland University Medical School, Homburg, Germany; ^8^Department of Dermatology, Medical Hospital of Quedlinburg, Quedlinburg, Germany; ^9^Department of Dermatology, Venereology, and Allergology, Helios St. Elisabeth Hospital Oberhausen, University of Witten-Herdecke, Oberhausen, Germany; ^10^Department of Dermatology, Venereology, and Allergology University Ulm, Ulm, Germany; ^11^Department of Dermatology, Elbe-Kliniken, Buxtehude, Germany; ^12^Skin Cancer Center, Department of Dermatology, Hannover Medical School, Hannover, Germany; ^13^Department of Dermatology, University Hospital Dresden, Dresden, Germany; ^14^Hautklinik, Klinikum der Stadt Ludwigshafen am Rhein gGmbH, Ludwigshafen, Germany; ^15^Department of Dermatology, University Hospital Kiel, Kiel, Germany; ^16^Translationale Hautkrebsforschung, University Medicine Essen, University of Duisburg-Essen, Essen, Germany

**Keywords:** PD-L1 quantification, melanoma, immune checkpoint blockade therapy, response, survival

## Abstract

**Background:**

PD-1-based immune checkpoint blockade (ICB) is a highly effective therapy in metastatic melanoma. However, 40-60% of patients are primarily resistant, with valid predictive biomarkers currently missing. This study investigated the digitally quantified tumor PD-L1 expression for ICB therapy outcome prediction.

**Patients and Methods:**

Tumor tissues taken prior to PD-1-based ICB for unresectable metastatic disease were collected within the prospective multicenter Tissue Registry in Melanoma (TRIM). PD-L1 expression (clone 28-8; cut-off=5%) was determined by digital and physician quantification, and correlated with therapy outcome (best overall response, BOR; progression-free survival, PFS; overall survival, OS).

**Results:**

Tissue samples from 156 patients were analyzed (anti-PD-1, n=115; anti-CTLA-4+anti-PD-1, n=41). Patients with PD-L1-positive tumors showed an improved response compared to patients with PD-L1-negative tumors, by digital (BOR 50.5% *versus* 32.2%; p=0.026) and physician (BOR 54.2% *versus* 36.6%; p=0.032) quantification. Tumor PD-L1 positivity was associated with a prolonged PFS and OS by either digital (PFS, 9.9 *versus* 4.6 months, p=0.021; OS, not reached *versus* 13.0 months, p=0.001) or physician (PFS, 10.6 *versus* 5.6 months, p=0.051; OS, not reached *versus* 15.6 months, p=0.011) quantification. Multivariable Cox regression revealed digital (PFS, HR=0.57, p=0.007; OS, HR=0.44, p=0.001) and physician (OS, HR=0.54, p=0.016) PD-L1 quantification as independent predictors of survival upon PD-1-based ICB. The combination of both methods identified a patient subgroup with particularly favorable therapy outcome (PFS, HR=0.53, p=0.011; OS, HR=0.47, p=0.008).

**Conclusion:**

Pre-treatment tumor PD-L1 positivity predicted a favorable outcome of PD-1-based ICB in melanoma. Herein, digital quantification was not inferior to physician quantification, and should be further validated for clinical use.

## Introduction

The introduction of immune checkpoint blockade (ICB) therapy led to a tremendous survival improvement in patients with advanced metastatic melanoma (1–3). PD-1-based ICB therapies can be used alone or in combination with CTLA-4 inhibitors (4). Despite improved long-term survival in responders, up to 60% of melanoma patients are primary resistant to PD-1-based ICB and have significantly inferior survival as a consequence (5). Approximately 40% of melanomas have a targetable tumor BRAF-V600 mutation with inhibition of the mitogen-activated protein kinase (MAPK) pathway as a viable alternative treatment option to ICB (6). There is a high need for valid pre-treatment biomarkers that predict the response to ICB to enable an optimal treatment choice for advanced melanoma patients. Nonspecific blood-based biomarkers have been reported to predict ICB treatment outcome such as serum lactate dehydrogenase (LDH) activity, as well as blood counts of lymphocytes and eosinophils (7–10). Tumor tissue-based biomarkers described to be associated with PD-1-based ICB therapy outcome are the density of tumor-infiltrating lymphocytes and the expression of PD-L1 (11). In particular, the quantification of PD-L1 expression in tumor tissue is widely used in routine clinical diagnostics of various cancer types such as non-small cell lung carcinoma (NSCLC), renal cell carcinoma, Hodgkin’s lymphoma, and colorectal carcinoma (12).

The role of tumor PD-L1 expression as a predictive biomarker in melanoma, however, is not clear, mainly because of the difficulties in evaluating melanomas with overall low PD-L1 expression and high melanin content (2, 13–15). Moreover, PD-L1 staining can be detected not only on the cell membrane but also intracellularly and shows high spatial heterogeneity, so its evaluation is associated with a high interobserver variability (16). In addition, it has already been shown that PD-L1 expression can differ greatly depending on the melanoma subtype and that melanoma subtypes respond differently to ICB (17). Whole-slide imaging and digital pathology have shown an improvement in the evaluation of the immunohistochemical tumor tissue stainings for HER2 and KI67 in breast cancer and for the Gleason classification in prostate cancer (18–20). In addition, a recent study demonstrated that a digital pathology algorithm can be helpful to the pathologist in the evaluation of tumor PD-L1 expression in melanin-bleached melanoma tissue samples. However, a correlation between digital PD-L1 quantification and therapy outcome has not been performed up to now (21).

The aim of the present study was to investigate digital PD-L1 quantification *versus* physician PD-L1 quantification in pre-treatment tumor tissue of melanoma patients as potential predictors of therapy outcome of a PD-1-based ICB.

## Patients and Methods

### Patients and Tissues

Formalin-fixed, paraffin-embedded (FFPE) tumor tissue samples from patients diagnosed with melanoma were prospectively collected within the multicenter translational study Tissue Registry in Melanoma (TRIM; CA209-578) performed within the framework of the skin cancer registry ADOREG of the German Dermatologic Cooperative Oncology Group (DeCOG). Out of this cohort, patients were selected for the present analysis according to the following criteria: Histologically confirmed diagnosis of melanoma of the skin, mucosa, or unknown primary; tumor tissue specimen obtained for analysis prior to a PD-1-based ICB for unresectable stage III or IV (AJCCv8) (18) metastatic disease; complete documentation of therapy outcome and follow-up, and availability of consecutive tissue slides stained for PD-L1 and control IgG, comparable in size and quality. Best overall response (BOR) was determined according to RECIST version 1.1 (22). Progression-free (PFS) and overall (OS) survival were defined as time from therapy start until disease progression or death, respectively; if no such event occurred, the date of the last patient contact was used as endpoint of survival assessment (censored observation). The study was approved by the ethics committee of the University Duisburg-Essen (15-6566-BO).

### PD-L1 Staining

The PD-L1 expression was assessed in FFPE tumor tissue specimens with the use of a rabbit monoclonal anti-human PD-L1 antibody (clone 28-8) and an analytically validated automated immunohistochemical assay (PD-L1 IHC 28-8 pharmDx for Autostainer Link 48; Dako, Agilent Technologies, Santa Clara, CA, USA), as described previously (23). A consecutive tissue slide of the same specimen was prepared accordingly for each sample, stained with non-specific IgG and used as negative control. Hematoxylin was used as nuclear staining. For detailed visualization of morphological structures, an additional control tissue slide was stained with hematoxylin and eosin (H&E).

### Quantification of PD-L1 Expression by the Physician

PD-L1 expression in tumor tissue was quantified as the percentage of live tumor cells that exhibited specific cell surface staining of any intensity in a section containing at least 100 evaluable tumor cells, with ≥5% defined as positive staining, as previously described (23). The cutoff >5% as the definition of PD-L1 positivity is recommended by the manufacturer of the assay and is the established standard in our department. This type of quantification of PD-L1 expression was performed by either pathologists or histopathologically experienced dermatologists or both using conventional bright field microscopy, and is referred to as “physician’s quantification”.

### Quantification of PD-L1 Expression by a Digital Algorithm

**Figure 1:**
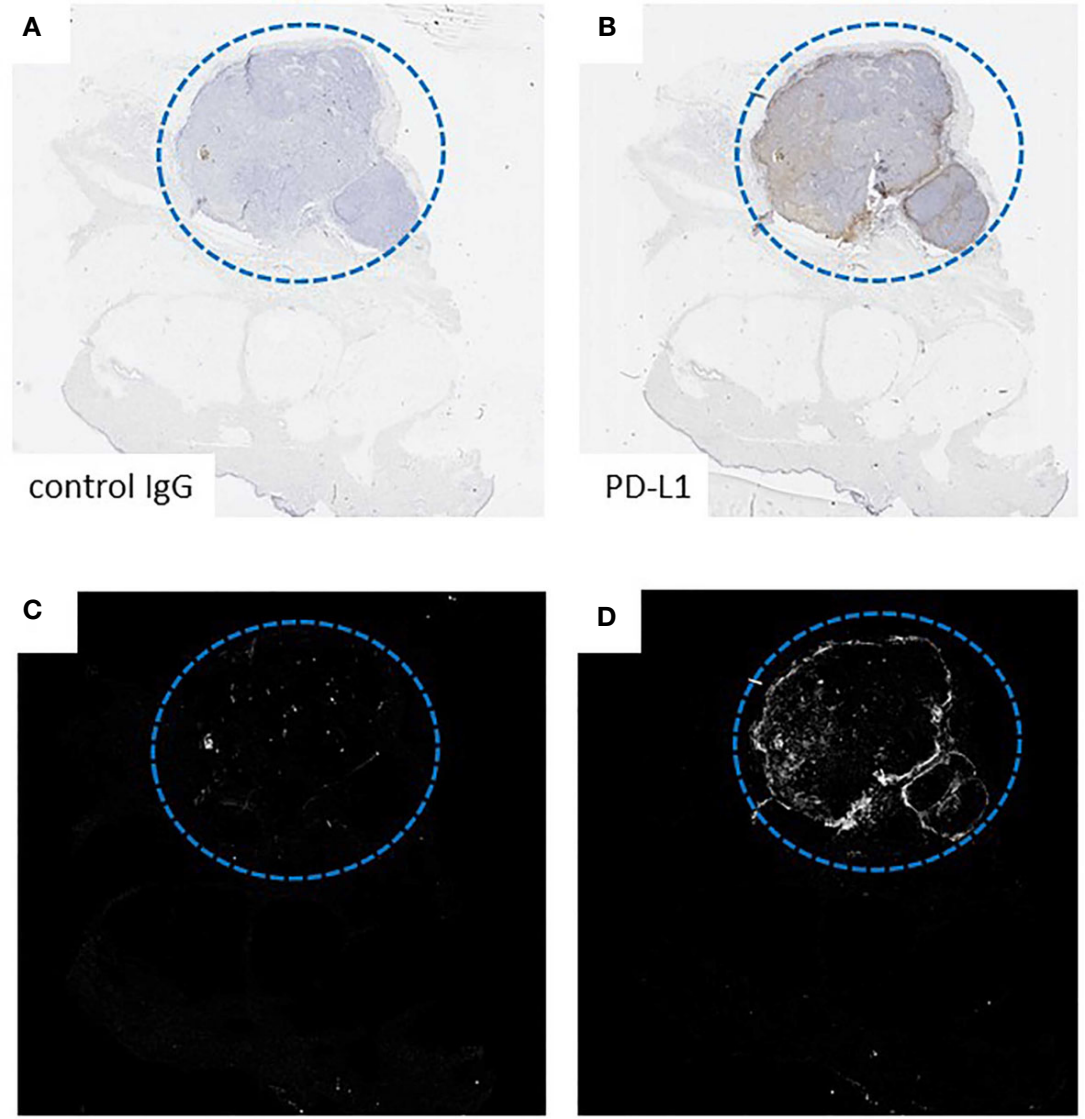
Exemplary presentation of the functioning of the digital algorithm on the basis of a sample from the patient group. Digital quantification of PD-L1 expression demonstrated on representative tissue slides from a subcutaneous melanoma metastasis. **(A, B)** Manual selection of the tumor regions of interest on an anti-PD-L1-stained slide and a consecutive negative control IgG-stained slide. **(C, D)** Binary masks of **(A, B)**.

The anti-PD-L1 stained slides and the negative control slides were digitized with the whole-slide scanner Aperio AT2 (Leica, Wetzlar, Germany) using a resolution of 20x. These digitized whole-slide images were used for the quantification of PD-L1 expression by a newly defined method based on a digital algorithm. This newly developed Java-based algorithm removes artifacts present on the tumor regions and quantifies the number of PD-L1 expressing cells. Corresponding tumor regions were manually selected as regions of interest on the anti-PD-L1-stained slide and the negative control slide; **Figures 1A, B**. Binary masks were generated by applying an intensity threshold for PD-L1 and melanin (brown) as well as hematoxylin (blue), **Figures 1C, D**. The binary masks were obtained by using various thresholding methods implemented by *Fiji* (https://imagej.net/Fiji), each best adapted to the type of signal. To generate the tissue masks (tumor and biopsy), the “Triangle” thresholding method was used. For the Melanin and PDL1 stainings,”MaxEntropy”, and “Moments” for the cellular nucleus. These methods are part of the Auto-threshold algorithms of Fiji and were chosen based on their accuracy (24). The digitized image of the respective negative control staining was used to deduct the background signal (melanin). The binary information for cellular and nuclear signals was co-registered and overlapping mask regions were used to extract the number of cells stained positive for PD-L1 (hematoxylin+/PD-L1+) or melanin (hematoxylin+/melanin+). In addition, the total number of cells (hematoxylin+) was determined to calculate the percentage of PD-L1+ cells relative to the total number of cells in the tumor area, with a percentage ≥5% defined as positive, similar to the physician evaluation. This method of PD-L1 quantification is referred to as “digital quantification”. It should be noted here that the digital algorithm quantifies not only PD-L1 expression of tumor cells, but of all cells in the ROI, such as macrophages or lymphocytes as well.

### Statistical Analysis

The survival endpoints (PFS and OS) were calculated using the Kaplan-Meier method for censored failure time data. The two-sided log-rank test was used to compare survival rates between groups. Multivariable analyses were performed using the Cox proportional hazards model. Known prognostic and predictive parameters of metastatic melanoma were included as covariates: age (≤65 *versus* >65 years), sex (male *versus* female), disease stage (III *versus* IV), location of primary (skin *versus* others), M category of metastasis (M1a/b *versus* M1c/d), LDH serum activity (elevated *versus* normal), therapy type (anti-PD-1 monotherapy *versus* anti-PD-1 plus anti-CTLA-4), BRAF mutation status (mutation *versus* wild type), and PD-L1 expression (positive *versus* negative). The correlation analysis was performed using the Pearson’s correlation coefficient. Best overall response (BOR) was calculated by chi-square test. P<0.05 was considered statistically significant. Survival analysis was performed with SPSS (Version 25, IBM, Armonk, NY, USA) and Graphpad Prism (Version 9, GraphPad Software, CA, USA).

## Results

### Patient Characteristics and Study Flow

**Figure 2:**
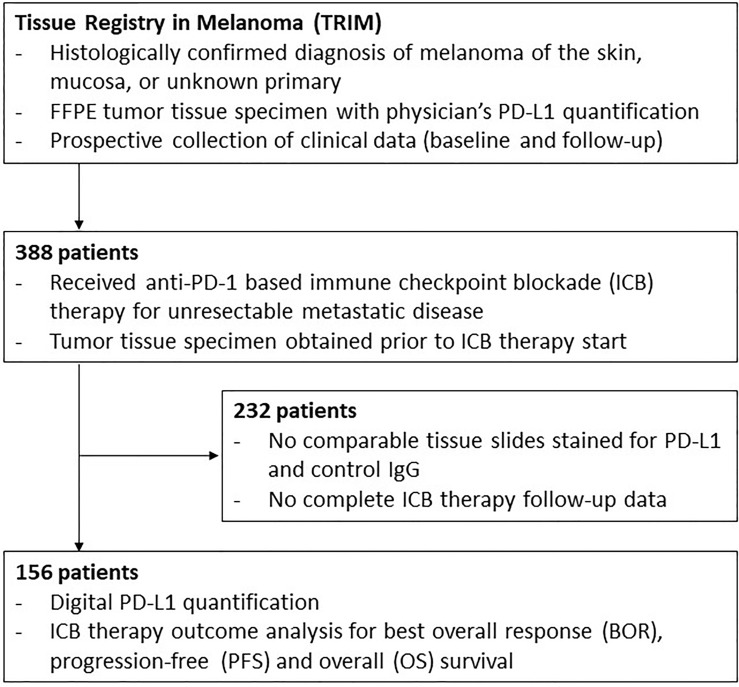
Study flow. Schematic presentation of the study flow. P-values <0.05 are in bold.

Of the patients participated in/were registered in the TRIM project, 388 patients started an anti-PD-1-based ICB therapy between February 2014 and July 2019 and 156 patients met the above mentioned selection criteria for the present study; **Figure 2**. The tumor tissue specimens examined for PD-L1 expression were obtained from primary tumors in 32/156 (20.5%) and from metastases in 124/156 (79.5%) of cases. 41/156 patients (26.2%) subsequently received treatment with anti-CTLA-4 plus anti-PD-1 ICB, and 115/156 patients (73.8%) received treatment with anti-PD-1 alone. At data cut-off (January 15, 2020) and after a median follow-up time of 26.4 months, patients showed a best objective response rate (BOR) (complete response, CR, plus partial response, PR) of 42.3% to anti-PD-1-based ICB. 74/156 patients (48.1%) died. For detailed clinical patient characteristics see, **Table 1**.

**Table 1:** Patient characteristics.

	*N (%)*
**Total**	156 (100%)
**Mean age (range)**	63 years (20 – 85 years)
**Sex**	
male	99 (63.5%)
female	57 (36.5%)
**Localization of primary**	
skin	140 (89.7%)
mucosa	2 (1.3%)
unknown primary	14 (9%)
**Type of melanoma**	
acral lentiginous melanoma	11 (7.1%)
lentigo malignant melanoma	2 (1.3%)
melanoma of unknown primary	14 (9.0%)
nodular malignant melanoma	57 (36.5%)
superficial spreading melanoma	26 (16.7%)
unclassifiable malignant melanoma	9 (5.8%)
unknown	37 (23.7%)
**BRAF mutation (tumor)**	
yes	60 (38.5%)
no	93 (59.6)
unknown	3 (1.9%
**AJCC stage and M category**	
III	21 (13.5%)
IV M1a	39 (25.0%)
IV M1b	28 (17.9%)
IV M1c	38 (24.4%)
IV M1d	30 (19.2%)
**Number of organs involved in metastasis**	
≤3	108 (69.2%)
>3	46 (29.5%)
unknown	2 (1.3%)
**LDH (serum)**	
normal (≤ULN)	104 (66.7%)
elevated (>ULN)	50 (32.1%)
unknown	2 (1.3%)
**ECOG performance status**	
0	123 (78.8%)
1	28 (17.9%)
>1	4 (2.6%)
unknown	1 (0.6%)
**Systemic pre-treatment**	
yes	48 (30.8%)
no	108 (69.2%)
**PD-1-based ICB therapy**	
PD-1 plus CTLA-4	41 (26.2%)
PD-1	115 (73.8%)

Characteristics of the investigated melanoma patient cohort at baseline of PD-1 based immune checkpoint blockade (ICB) therapy. Disease staging was performed according to AJCCv8. LDH, lactate dehydrogenase; ULN, upper limit of normal.

### Comparison of PD-L1 Quantification by the Physician and the Digital Algorithm Shows a Concordant Result in Over 60% of Cases

**Figure 3:**
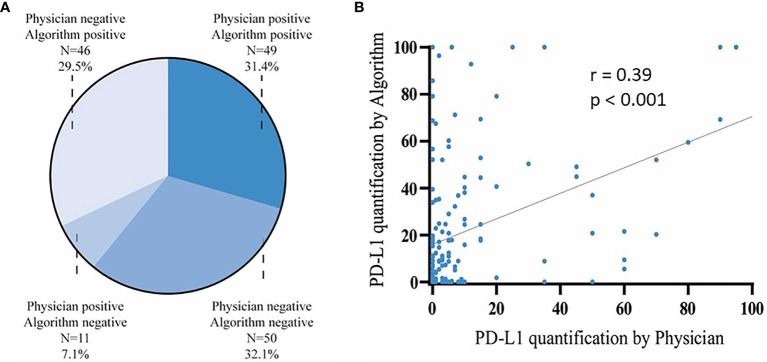
Comparison of PD-L1 calculation by physician and digital algorithm. **(A)** Distribution of PD-L1 quantification in tumor tissue specimen of n=156 melanoma patients by the physician and the digital algorithm. **(B)** Correlation of PD-L1 quantification by the physician (x axis) *versus* the digital algorithm (y axis) in n=156 patients (Pearson’s correlation; r = 0.39; p < 0.001).

To investigate whether there was comparability between the two methods, we first examined how many patients were scored the same by the physician and by the digital algorithm with respect to PD-L1 positivity. Physician’s *versus* digital quantification was PD-L1 positive in 38.5% (n=60/156) *versus* 60.9% of cases (n=95/156), respectively. The PD-L1 quantification of the tumor specimens by the physician and the digital algorithm showed the same result in terms of positivity *versus* negativity in 99 (63.5%) of the analyzed patients, with 49 tumors (31.4%) classified as PD-L1 positive and 50 tumors (32.1%) as PD-L1 negative. 57 tumors (36.5%) were scored differently by the physician *versus* the digital algorithm, with 46 tumors (29.4%) scored as PD-L1 positive by the digital algorithm only and 11 tumors (7.1%) scored as PD-L1 positive by the physician only; **Figure 3A**. The PD-L1 quantification by the physician and the digital algorithm showed a significant correlation (Pearson’s correlation; r = 0.39; p <0.001; **Figure 3B**). In summary, 60.3% of patients showed the same assessment regarding PD-L1 positivity by physician and digital algorithm. In the cases that were classified differently by both measurement methods, the digital algorithm showed PD-L1 positive findings more frequently.

### Tumor PD-L1 Positivity by Physician’s Quantification Predicts Favorable Outcome of Anti-PD-1-Based ICB Therapy

**Figure 4:**
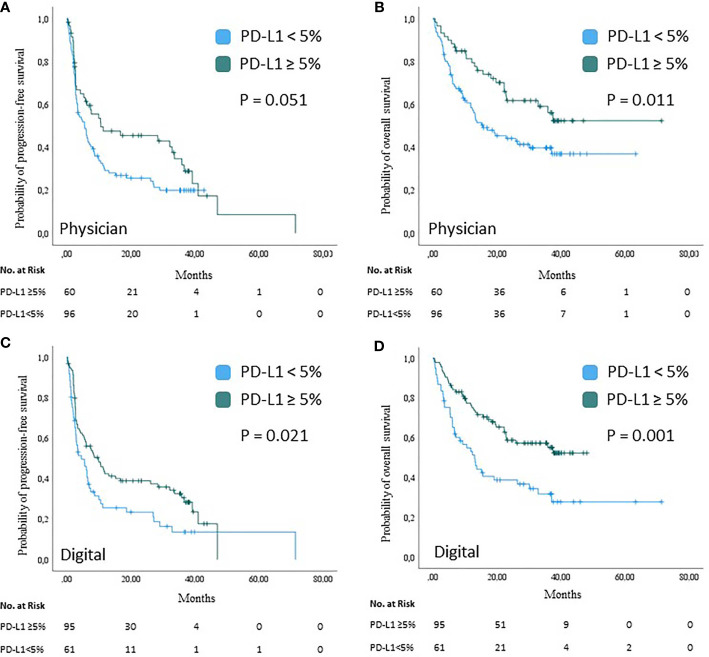
Survival analysis based on PD-L1 expression analysis by physician or digital algorithm. Kaplan-Meier curves showing the probability of progression-free **(A, C)** and overall **(B, D)** survival of n=156 melanoma patients upon treatment with PD-1-based immune checkpoint blockade by tumor PD-L1 expression. Tumor PD-L1 expression was assessed by physician’s quantification **(A, B)** and digital quantification **(C, D)**, respectively. Censored observations are indicated by vertical bars; P values were calculated using the log-rank test.

We next investigated the feasibility of physician PD-L1 analysis, traditionally used in the clinic, to predict patient response to therapy and survival. Melanoma patients with PD-L1 positive tumors by physician’s quantification (n=60; 38.5%) showed an improved therapy response upon anti-PD-1-based ICB (BOR=54.2%) as compared to patients with PD-L1 negative tumors (n=96; BOR=36.6%; p=0.032). The median PFS after start of anti-PD-1-based ICB in patients with PD-L1 positive tumors by physician’s quantification was 10.6 months (95% CI=0–32.6 months); the median OS was not reached. In patients with PD-L1 negative tumors by physician’s quantification the median PFS was 5.6 months (95% CI=3.0–8.1 months), and the median OS was 15.6 months (95% CI=6.4–24.8 months). Survival differences between PD-L1 positive and negative tumors by physician’s quantification showed borderline significance for PFS (P=0.051), and strong significance for OS (P=0.011); **Figures 4A, B**. A multivariable Cox regression analysis was performed to evaluate the predictive value of tumor PD-L1 expression by physician’s quantification for the survival outcome of anti-PD-1-based ICB therapy in metastatic melanoma. Tumor PD-L1 expression by physician quantification was not an independent predictor of PFS (HR= 0.7; 95% CI=0.46–1.06; P=0.094), but of OS (HR=0.54; 95% CI=0.33-0.89; P=0.016); **Supplementary Table 1**. None of the other parameters tested was independently predictive for survival upon anti-PD-1-based ICB therapy. In conclusion, PD-L1 expression analysis conventionally used in the clinic can be used in melanoma by physicians to predict treatment response and patient survival under ICB.

### Tumor PD-L1 Positivity by Digital Quantification Predicts Favorable Outcome of Anti-PD-1-Based ICB Therapy

The next step was to investigate whether the newly programmed digital algorithm was also suitable for PD-L1 analysis. Melanoma patients with PD-L1 positive tumors by digital quantification (n=95; 60.9%) showed an improved therapy response upon anti-PD-1 based ICB (BOR=50.5%) as compared to patients with PD-L1 negative tumors (n=61; BOR=32.2%; p=0.026). In patients with PD-L1 positive tumors by digital quantification the median PFS was 9.9 months (95% CI=5.2–14.7 months); the median OS was not reached. In patients with PD-L1 negative tumors by digital quantification the median PFS was 4.6 months (95% CI=1.4–7.8 months), and the median OS was 13.0 months (95% CI=8.6–17.6 months). Survival upon anti-PD-1-based ICB therapy was significantly longer in patients with PD-L1 positive tumors by digital quantification than in patients with PD-L1 negative tumors (PFS, P=0.021; OS, P=0.001); **Figures 4C, D**. A multivariable Cox regression analysis was performed to evaluate the predictive value of tumor PD-L1 expression by digital quantification under consideration of the same prognostic and predictive parameters as mentioned above. Among the parameters tested, the PD-L1 expression by digital quantification proved to be the only independent predictor of survival (PFS, HR=0.57, 95% CI=0.37–0.86, P=0.007; OS, HR=0.44, 95% CI=0.27–0.7, P=0.001); **Supplementary Table 2**. In conclusion, it was shown that the digital algorithm is also suitable to estimate treatment response and patient survival under ICB by PD-L1 expression analysis.

### Tumors Showing PD-L1 Positivity by Both Physician’s and Digital Quantification Are Associated With a Particularly Favorable Therapy Outcome

**Figure 5:**
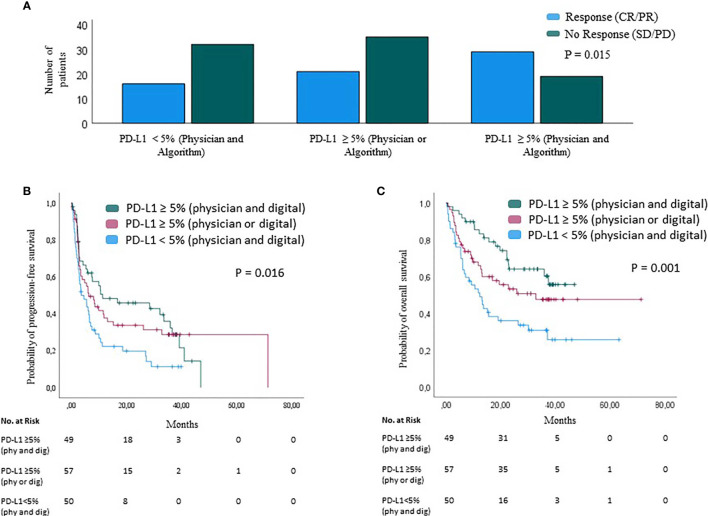
Therapy response and survival analysis based on PD-L1 expression analysis by physician and digital algorithm. Best overall response, BOR **(A)** and survival **(B, C)** of n=156 melanoma patients upon PD-1-based immune checkpoint inhibition by tumor PD-L1 expression. Tumor PD-L1 expression is presented as a combination of physician and digital quantification. **(A)** BOR is highest in patients with tumor PD-L1 positivity by both physician and digital quantification (CR/PR=60.4%; right), compared to patients with tumor PD-L1 positivity by only one of both quantification methods (CR/PR=37.5%; center), and patients whose tumors are classified as PD-L1 negative by both physician and digital quantification (CR/PR=33.4%; left); Chi-square test P = 0.015. **(B, C)** Progression-free **(B)** and overall survival **(C)** by tumor PD-L1 expression combined of physician and digital quantification. P values were calculated using the log-rank test.

Finally, it was investigated whether the combination of both measurement methods can be used to further improve the response to therapy and patient survival. Patients with tumors classified as PD-L1-positive by both methods, physician and digital quantification, showed the highest therapy response to anti-PD1-based ICB (BOR=60.4) compared with patients with tumors assessed as PD-L1-positive by only one method (BOR=37.5%) or with patients with tumors assessed as PD-L1-negative by both methods (BOR=33.4%) (P=0.015) (**Figure 5A**), the median PFS was 11.4 months (95% CI=0-33 months) while the median OS was not reached. Patients with tumors rated PD-L1 positive only by the physician or by the digital algorithm (n=57) had a median PFS of 6.4 months (95% CI=2.5-8.7 months); the median OS was 32.9 months. Patients with tumors classified as PD-L1 negative by both quantification methods (n=50) had a median PFS of 3.6 months (95% CI=0.7-6.5 months) and a median OS of 12.4 months (95% CI=7.1-17.7 months). Thus, tumors classified as PD-L1 positive by both the physician and the digital algorithm are associated with a significant prolongation of the patient’s survival upon anti-PD-1-based ICB therapy (PFS, P= 0.016; OS, P=0.001); **Figures 5B, C**. In the multivariable Cox regression analysis using the same cofactors as described above, tumor PD-L1 positivity by both quantification methods independently predicted a favorable PFS (HR=0.53, 95%-CI=0.32–0.86, p=0.011) and OS (HR=0.47, 95%-CI=0.27–0.82, p=0.008) of the respective patients; **Table 2**. Patients whose tumors were tested positive only by the physician or by the digital algorithm showed no relevant differences with regard to PFS and OS as compared to each other. The addition of PD-L1 expression analysis by the digital algorithm to conventional physician analysis has greatly improved the predictive power of PD-L1 analysis in terms of response to therapy and patient survival.

**Table 2:** Multivariable cox regression analysis (combined physician’s and digital PD-L1 quantification).

Parameters included	PFS		OS	
	Hazard ratio (95% CI)	*p*-value	Hazard ratio (95% CI)	*p*-value
**Age**	0.69 (0.43 – 1.09)	0.11	0.92 (0.56 – 1.51)	0.74
(≤65 *versus >*65 years)				
**Disease stage**	1.58 (0.76 – 3.25)	0.22	1.34 (0.56 – 3.17)	0.50
(III *versus* IV)				
**Localization of primary**	1.49 (0.35 – 6.42)	0.59	0.56 (0.08 – 4.22)	0.58
(skin *versus* other)				
**Serum LDH**	0.83 (0.52 -1.32)	0.44	1.05 (0.64 - 1.71)	0.86
(elevated *versus* normal)				
**Therapy type**	0.70 (0.41 – 1.20)	0.19	0.87 (0.49 – 1.54)	0.64
(single agent anti-PD-1 *versus* anti-PD-1 plus anti-CTLA-4)				
**M category of metastasis**	0.77 (0.47 – 1.27)	0.30	1.15 (0.64 - 2.05)	0.64
(M1a or b *versus* M1c)				
**Gender**	1.13 (0.73 – 1.75)	0.57	0.90 (0.54 - 1.48)	0.669
(male versus female)				
**BRAF status**	1.06 (0.67 – 1.66)	0.82	0.92 (0.55 - 1.51)	0.73
(mutation versus wildtype)				
**Tumor PD-L1 expression by physician’s and algorithm’s quantification**	0.53 (0.32 – 0.86)	**0.011**	0.47 (0.27 – 0.82)	**0.008**
(positive versus negative; cut-off ≥5%)				

Multivariable Cox regression of the combined PD-L1 analysis by physician and digital algorithm including clinical and molecular parameters determined at the start of anti-PD-1 therapy in n=156 patients.

P-values <0.05 are in bold.

## Discussion

Immunohistochemical PD-L1 expression analysis has been shown to be a predictive biomarker for ICB treatment outcomes in numerous tumor entities and, in this regard, is already routinely being considered for treatment decisions in entities such as NSCLC and urogenitary cancers (12). However, in melanoma the role of PD-L1 expression as a predictive biomarker for therapy outcome is currently under debate and has still not entered the clinical routine for treatment decision making. Herein, one major reason is the high inter-observer variability of PD-L1 quantification reported for melanoma, mainly due to melanin pigmentation hampering the evaluation process. To overcome these hurdles, we here investigated the association between pre-treatment tumor PD-L1 expression and ICB therapy outcome using two independent methods of PD-L1 expression quantification, one by trained physicians and the other by a newly proposed digital algorithm. The digital quantification method harbors the advantage to be applicable regardless of the presence of melanin pigmentation. Our results showed a prolonged PFS and OS in melanoma patients whose tumors were classified as PD-L1 positive by both methods of PD-L1 quantification, with the digital quantification not being inferior to the physician quantification.

The positive correlation of pre-treatment tumor PD-L1 expression with PFS and OS observed in this study is consistent with previously published data from clinical trials in metastatic melanoma (2, 25, 26). Interestingly, the combination of PD-L1 quantification methods, the physician’s and the digital algorithm method, showed the longest survival for patients with PD-L1 positive tumors with a median PFS of 11.4 months and a median OS not reached, and proved to be independently predictive by multivariable testing. In the existing literature, tumor PD-L1 expression in melanoma is considered to play the role of a prognostic marker but to have little pre-therapeutic predictive value (27). In contrast, our analysis of pre-treatment tumor tissue samples shows that melanoma patients whose tumors were evaluated as PD-L1 positive by both the physicians and the digital algorithm had a BOR on ICB of 60.4%, indicating that tissue PD-L1 expression has predictive value.

Currently, targeted therapy with BRAF/MEK inhibition is available for melanoma patients with BRAF mutation as an alternative or an addition to ICB (28–30). In these patients, whose tumors harbor a targetable BRAF mutation, there is a lack of predictive biomarkers that help to choose the optimal individualized therapy. Here, tumor PD-L1 expression quantification could be a helpful tool, assuming that patients showing PD-L1 positivity are more likely to benefit from ICB, and patients with PD-L1-negative tumors may be more likely to benefit from targeted therapy.

Obviously, the use of PD-L1 as a biomarker is difficult, as different cut-offs and a high intratumoral heterogeneity with dynamic changes exist as well as different scoring systems are available. A total of four different scoring systems are established and in clinical use for tumor PD-L1 quantification. For melanoma, the most commonly used scoring system is the Tumor Proportion Score (TPS), which restrictively quantifies only tumor cells that exhibit linear staining of the membrane. Other cancer entities for which the TPS is used include NSCLC and carcinomas of the head and neck. The combined positive score (CPS), which quantifies tumor cells and immune mononuclear cells, is used for urothelial and gastric carcinomas. In urothelial carcinoma, the immune cell score (IC), which quantifies all immune cells stained for PD-L1, is in use. In the melanoma score (MEL score), PDL-1-positive mononuclear immune cells and tumor cells are quantified, similar to the CPS (16, 31, 32). In the present study, the physicians used the restrictive TPS, whereas the digital algorithm used the CPS as classification system that includes PD-L1 expression on associated immune cells. Indeed, these scoring differences explain why the digital quantification showed higher frequencies of PD-L1 positivity compared to the physician’s quantification. Interestingly, 63.5% of cases still showed the same result in terms of positivity or negativity.

Since the present algorithm is a pixel-based image analysis algorithm, its application is not limited to PD-L1 analysis and can be readily used for other immunohistochemical staining for quantification in clinical and research settings. In addition, the digital algorithm can also be used for quantification for multiplex immunofluorescence imaging in translational and basic research.

The present study and the digital PD-L1 quantification method also unraveled some limitations. The digital algorithm currently is only a semi-quantitative measuring tool, as the physician still has to select the target tumor areas to be analyzed. In contrast to physician quantification with the currently recommended method TPS, where only tumor cells with PD-L1 membrane staining are counted, the digital algorithm quantifies all cells of the tumor microenvironment and does not distinguish between cytosol staining, nuclear staining or membrane staining similar to CPS. Thus, in the present study, two different investigators (physician *vs.* digital algorithm) were compared which used different scoring systems for quantification (TPS *vs.* CPS). Further modifications, e.g. the addition of artificial intelligence technologies, are required to transform the actual digital algorithm into a measuring instrument that is completely independent from the physician. Another limitation of this study is that we did not analyze melanoma samples from patients who were treated with anti-CTLA-4 monotherapy and therefore cannot conclude to what extent PD-L1 expression plays a role in these patients. However, it must be noted that anti-CTLA-4 monotherapy alone plays almost no role in melanoma therapy any longer. Notably, a large proportion of patients were pre-treated, including BRAF-mutated patients with BRAF inhibitors, which may have influenced the results on treatment efficacy and identification and validation of PD-L1 as a predictive biomarker. In conclusion, our results demonstrate that pre-treatment tumor PD-L1 quantification by a digital algorithm is not inferior to the quantification by physicians as predictors of ICB therapy outcome. Moreover, the combination of both quantification methods significantly improved the predictive value. Accordingly, a digital quantification of tumor PD-L1 expression could facilitate diagnostic procedures, and improve the prediction of treatment outcomes at treatment decision making in patients with metastatic melanoma. Further studies are planned to investigate tumor PD-L1 expression by different methods in melanoma patients treated in the adjuvant setting.

## Data Availability Statement

The raw data supporting the conclusions of this article will be made available by the authors, without undue reservation.

## Ethics Statement

The studies involving human participants were reviewed and approved by Ethics committee of the University Duisburg-Essen. The patients/participants provided their written informed consent to participate in this study.

## Author Contributions

J-MP, CS, DE, and SU did the study design. Digital algorithm was designed by CS and DE. J-MP, RH, PT, JeU, ClP, JoU, AK, ChP, PM, RG, FM, ED, MW, LZ, EL, JB, GL, AR, DS, and SU contributed patient data. J-MP, KG, EH, and SU performed the physician’s PD-L1 quantification. All authors contributed to the article and approved the submitted version.

## Funding

This work was in part supported by Bristol Myers Squibb for the multicenter translational study “Tissue Registry in Melanoma” (TRIM) within the framework of the skin cancer registry ADOREG of the German Dermatologic Cooperative Oncology Group (DeCOG).

## Conflict of Interest

J-MP served as consultant and/or has received honoraria from Bristol-Myers Squibb, Novartis and received travel support from Bristol-Myers Squibb, Novartis and Therakos. PT declares Invited Speaker´s honoraria from Bristol-Myers Squibb, Novartis, MSD, Pierre-Fabre, CureVac, Roche, Kyowa Kirin, Biofrontera, Advisory Board honoraria from Bristol-Myers Squibb, Novartis, Pierre-Fabre, Merck Serono, Sanofi, Roche, Kyowa Kirin, and Travel support from Bristol-Myers Squibb, and Pierre-Fabre. JoU is on the advisory board or has received honoraria and travel support from Amgen, Bristol Myers Squibb, GSK, LeoPharma, Merck Sharp and Dohme, Novartis, Pierre Fabre, Roche, Sanofi outside the submitted work. ClP received honoraria (speaker honoraria or honoraria as a consultant) and travel support from: Novartis, BMS, Roche, Merck Serono, MSD, Celgene, AbbVie, AMGEN, SUNPHARMA, Allergy Therapeutics and LEO. LZ served as consultant and/or has received honoraria from Roche, Bristol-Myers Squibb, Merck Sharp & Dohme, Novartis, Pierre-Fabre, and Sanofi; Research funding to institution: Novartis; travel support from Merck Sharp & Dohme, Bristol-Myers Squibb, Amgen, Pierre-Fabre, and Novartis, outside the submitted work. EL served as consultant and/or has received honoraria from Amgen, Actelion, Roche, Bris-tol-Myers Squibb, Merck Sharp & Dohme, Novartis, Janssen, Medac, Sanofi, Sunpharma and travel support from Amgen, Merck Sharp & Dohme, Bristol-Myers Squibb, Amgen, Pierre Fabre, Sunpharma and Novartis, outside the submitted work. JB is receiving speaker’s bureau honoraria from Amgen, Pfizer, MerckSerono, Recordati and Sanofi, is a paid consultant/advisory board member/DSMB member for 4SC, Almirall, Boehringer Ingelheim, ICON, InProTher, MerckSerono, Pfizer, and Sanofi/Regeneron. His group receives research grants from Merck Serono, HTG, IQVIA, and Alcedis. GL has received travel support from Sun Pharma. AR reported grants from Novartis, Bristol Myers Squibb, and Adtec; personal fees from Merck Sharp & Dohme; and nonfinancial support from Amgen, Roche, Merck Sharp & Dohme, Novartis, Bristol Myers Squibb, and Teva. DS received grants and other support from Bristol-Myers Squibb, personal fees from Bristol-Myers Squibb during the conduct of the study; personal fees from Amgen; personal fees from Boehringer Ingelheim; personal fees from InFlarX; personal fees and other support from Roche; grants, personal fees and other support from Novartis; personal fees from Incyte; personal fees and other support from Regeneron; personal fees from 4SC; personal fees from Sanofi; personal fees from Neracare; personal fees from Pierre-Fabre; personal fees and other support from Merck-EMD; personal fees from Pfizer; personal fees and other support from Philiogen; personal fees from Array, personal fees and other support from MSD Sharp & Dohme, outside the submitted work. SU declares research support from Bristol Myers Squibb and Merck Serono; speakers and advisory board honoraria from Bristol Myers Squibb, Merck Sharp & Dohme, Merck Serono, Novartis and Roche, and travel support from Bristol Myers Squibb, and Merck Sharp & Dohme.

The remaining authors declare that the research was conducted in the absence of any commercial or financial relationships that could be construed as a potential conflict of interest.

The authors declare that this study received funding from Bristol Myers Squibb. The funder had the following involvement with the study: Financing of test material.

## Publisher’s Note

All claims expressed in this article are solely those of the authors and do not necessarily represent those of their affiliated organizations, or those of the publisher, the editors and the reviewers. Any product that may be evaluated in this article, or claim that may be made by its manufacturer, is not guaranteed or endorsed by the publisher.

## Supplementary Material

Supplementary Table 1 Multivariable Cox regression analysis (physician PD-L1 quantification) . Multivariable Cox regression analysis of tumor PD-L1 expression by physician quantification and covariates at baseline of anti-PD-1 ICB therapy in *n=*156 melanoma patients.

Supplementary Table 2 Multivariable Cox regression analysis (digital PD-L1 quantification). Multivariable Cox regression analysis of tumor PD-L1 expression by digital quantification and covariates at baseline of anti-PD-1 ICB therapy in n=156 melanoma patients.
