# Supplementary material for: Digital Quantification of Tumor PD-L1 Predicts Outcome of PD-1-Based Immune Checkpoint Therapy in Metastatic Melanoma
Source: Front Oncol. 2021 Sep 21;11:741993. doi: 10.3389/fonc.2021.741993 (PMC8491983; doi:10.3389/fonc.2021.741993)
Supplement: Supplementary Table 1 — Multivariable Cox regression analysis (physician PD-L1 quantification) . Multivariable Cox regression analysis of tumor PD-L1 expression by physician quantification and covariates at baseline of anti-PD-1 ICB therapy in n=156 melanoma patients. [file Table_1.docx]

Supplementary Table 1. Multivariable Cox regression analysis (physician PD-L1 quantification)

|  | **PFS** | | | **OS** | |
| --- | --- | --- | --- | --- | --- |
| **Parameters included** | **Hazard ratio (95% CI)** | ***p*-value** | **Hazard ratio (95% CI)** | | ***p*-value** |
| **Age** | 0.72 (0.47 - 1.11) | 0.14 | 0.94 (0.58 - 1.55) | | 0.82 |
| (≤65 *versus* >65 years) |  |  |  |  |  |
| **Disease stage** | 1.35 (0.67 – 2.71) | 0.41 | 1.37 (0.58 - 3.25) | | 0.47 |
| (III *versus* IV) |  |  |  |  |  |
| **Localisation of primary** | 1.34 (0.31 - 5.72) | 0.70 | 0.55 (0.73 - 4.13) | | 0.56 |
| (skin *versus* other) |  |  |  |  |  |
| **Serum LDH** | 0.96 (0.63 – 1.48) | 0.86 | 1.09 (0.67 - 1.78) | | 0.71 |
| (elevated *versus* normal) |  |  |  |  |  |
| **Therapy type** | 0.74 (0.45 - 1.21) | 0.23 | 0.86 (0.49 - 1.53) | | 0.61 |
| (single agent anti-PD-1 *versus* anti-PD-1 plus anti-CTLA-4) |  |  |  |  |  |
| **M category of metastasis** | 0.92 (0.57 – 1.49) | 0.73 | 1.15 (0.65 - 2.05) | | 0.64 |
| (M1a or b *versus* M1c) |  |  |  |  |  |
| **Sex** | 1.15 ( 0.77 - 1.73) | 0.50 | 0.92 (0.56 - 1.51) | | 0.73 |
| (male *versus* female) |  |  |  |  |  |
|  |  |  |  |  |  |
| **BRAF status** | 0.95 ( 0.62 – 1.45) | 0.81 | 0.94 (0.57 - 1.54) | | 0.80 |
| (mutation *versus* wildtype) |  |  |  |  |  |
| **Tumor PD-L1 expression by physician’s quantification** | 0.70 (0.46 – 1.063) | 0.094 | 0.54 (0.33 - 0.89) | | **0.016** |
| (positive *versus* negative; cut-off ≥5%) |  |  |  |  |  |

Multivariable Cox regression analysis of tumor PD-L1 expression by physician quantification and covariates at baseline of anti-PD-1 ICB therapy in *n=*156 melanoma patients. P values <0.05 are in bold
