# Supplementary material for: Digital Quantification of Tumor PD-L1 Predicts Outcome of PD-1-Based Immune Checkpoint Therapy in Metastatic Melanoma
Source: Front Oncol. 2021 Sep 21;11:741993. doi: 10.3389/fonc.2021.741993 (PMC8491983; doi:10.3389/fonc.2021.741993)
Supplement: Supplementary Table 2 — Multivariable Cox regression analysis (digital PD-L1 quantification). Multivariable Cox regression analysis of tumor PD-L1 expression by digital quantification and covariates at baseline of anti-PD-1 ICB therapy in n=156 melanoma patients. [file Table_2.docx]

Supplementary Table 2. Multivariable Cox regression analysis (digital PD-L1 quantification)

|  | **PFS** | | **OS** | |
| --- | --- | --- | --- | --- |
| **Parameters included** | **Hazard ratio (95% CI)** | ***p*-value** | **Hazard ratio (95% CI)** | ***p*-value** |
| **Age** | 0.71 (0.47 - 1.10) | 0.12 | 0.93 (0.57 - 1.54) | 0.79 |
| (≤65 *versus* >65 years) |  |  |  |  |
| **Disease stage** | 1.36 (0.68 – 2.71) | 0.39 | 1.35(0.53 - 2.94) | 0.60 |
| (III *versus* IV) |  |  |  |  |
| **Localisation of primary** | 1.83 (0.43 – 7.82) | 0.42 | 0.91 (0.12 - 6.88) | 0.93 |
| (skin *versus* other) |  |  |  |  |
| **Serum LDH** | 0.87 (0.56 - 1.35) | 0.53 | 0.97 (0.59 - 1.60) | 0.91 |
| (elevated *versus* normal) |  |  |  |  |
| **Therapy type** | 0.73 (0.44 - 1.19) | 0.20 | 0.85 (0.48 - 1.50) | 0.57 |
| (single agent anti-PD-1 *versus* anti-PD-1 plus anti-CTLA-4) |  |  |  |  |
| **M category of metastasis** | 0.89 (0.55 - 1.43) | 0.62 | 1.23 (0.69 - 2.21) | 0.48 |
| (M1a or b *versus* M1c) |  |  |  |  |
| **Gender** | 1.13 (0.75 - 1.70) | 0.56 | 0.90 (0.54 - 1.48) | 0.67 |
| (male *versus* female) |  |  |  |  |
|  |  |  |  |  |
| **BRAF status** | 0.90 (0.59 – 1.38) | 0.63 | 0.87 (0.53 - 1.44) | 0.57 |
| (mutation *versus* wildtype) |  |  |  |  |
| **Tumor PD-L1 expression by algortihm’s quantification** | 0.57 (0.37 - 0.86) | **0.007** | 0.44 (0.57 - 0.70) | **0.001** |
| (positive *versus* negative; cut-off ≥5%) |  |  |  |  |

Multivariable Cox regression analysis of tumor PD-L1 expression by digital quantification and covariates at baseline of anti-PD-1 ICB therapy in n=156 melanoma patients. P values <0.05 are in bold
